# NADPH oxidase and reactive oxygen species contribute to alcohol-induced microglial activation and neurodegeneration

**DOI:** 10.1186/1742-2094-9-5

**Published:** 2012-01-12

**Authors:** Liya Qin, Fulton T Crews

**Affiliations:** 1 Bowles Center for Alcohol Studies, School of Medicine, CB#7178 UNC-CH, Chapel Hill, NC 27599, USA

**Keywords:** NADPH oxidase, Reactive oxygen species, neuroinflammation, neurodegeneration

## Abstract

**Background:**

Activation of microglia causes the production of proinflammatory factors and upregulation of NADPH oxidase (NOX) that form reactive oxygen species (ROS) that lead to neurodegeneration. Previously, we reported that 10 daily doses of ethanol treatment induced innate immune genes in brain. In the present study, we investigate the effects of chronic ethanol on activation of NOX and release of ROS, and their contribution to ethanol neurotoxicity.

**Methods:**

Male C57BL/6 and NF-κB enhanced GFP mice were treated intragastrically with water or ethanol (5 g/kg, i.g., 25% ethanol w/v) daily for 10 days. The effects of chronic ethanol on cell death markers (activated caspase-3 and Fluoro-Jade B), microglial morphology, NOX, ROS and NF-κB were examined using real-time PCR, immunohistochemistry and hydroethidine histochemistry. Also, Fluoro-Jade B staining and NOX gp91^phox ^immunohistochemistry were performed in the orbitofrontal cortex (OFC) of human postmortem alcoholic brain and human moderate drinking control brain.

**Results:**

Ethanol treatment of C57BL/6 mice showed increased markers of neuronal death: activated caspase-3 and Fluoro-Jade B positive staining with Neu-N (a neuronal marker) labeling in cortex and dentate gyrus. The OFC of human post-mortem alcoholic brain also showed significantly more Fluoro-Jade B positive cells colocalized with Neu-N, a neuronal marker, compared to the OFC of human moderate drinking control brain, suggesting increased neuronal death in the OFC of human alcoholic brain. Iba1 and GFAP immunohistochemistry showed activated morphology of microglia and astrocytes in ethanol-treated mouse brain. Ethanol treatment increased NF-κB transcription and increased NOX gp91^phox ^at 24 hr after the last ethanol treatment that remained elevated at 1 week. The OFC of human postmortem alcoholic brain also had significant increases in the number of gp91^phox ^+ immunoreactive (IR) cells that are colocalized with neuronal, microglial and astrocyte markers. In mouse brain ethanol increased gp91^phox ^expression coincided with increased production of O_2_^- ^and O_2_^- ^- derived oxidants. Diphenyleneiodonium (DPI), a NOX inhibitor, reduced markers of neurodegeneration, ROS and microglial activation.

**Conclusions:**

Ethanol activation of microglia and astrocytes, induction of NOX and production of ROS contribute to chronic ethanol-induced neurotoxicity. NOX-ROS and NF-κB signaling pathways play important roles in chronic ethanol-induced neuroinflammation and neurodegeneration.

## Background

Alcohol can cause brain damage [1] and lead to neurodegeneration in some cases [2]. Alcoholics, likely due to heavy alcohol consumption have reduced brain mass, cortical neuronal loss, other neuropathological changes as well as impaired cognitive functions and mild dementia [3-5]. Binge ethanol administration in adult rats is known to cause brain damage reduced by anti-oxidants [6-8]. However, the mechanism of ethanol induced neurodegeneration is uncertain. Previous work from our laboratory found 10 daily doses of ethanol treatment to male mice induced microglial activation, increased proinflammatory cytokines (TNFα, IL-1β, IL-6 etc.) and chemokines (MCP-1) and up-regulated NOX, resulting in production of ROS [9]. We also found increased microglial markers and levels of the chemokine, MCP-1, in post-mortem human alcoholic brain [10]. Others studying female mice following 5 months of ethanol drinking found chronic ethanol activation of nuclear factor kappa-B (NF-κB) pathways, markers of increased microglia and astrocyte activation, induction of the proinflammatory oxidases, inducible nitric oxide synthase, and cyclo-oxygenase COX-2, as well as increased cytokine levels in the cerebral cortex that were related to increased activated caspase-3, a marker of cell death [11]. In the present study, in male mice, we investigate NF-κB, NADPH oxidase (NOX) and ROS involvement in neuronal damage.

NF-κB is a transcription factor that in brain is involved in proinflammatory gene activation in glia as well as other gene regulation. Acute ethanol treatment of rats activates NF-κB in the brain [12]. Chronic binge ethanol treatment of rats causes neuronal degeneration and increased NF-κB-DNA binding, with both being reversed by inhibition of NF-κB-DNA binding [13]. Ethanol treatment of brain slice cultures has been found to increase multiple NF-κB proinflammatory target genes [14,15]. However, the relationship between proinflammatory gene induction and neuronal death is not clearly understood. Activation of glial cells, especially microglia, that release pro-inflammatory factors and reactive oxygen species (ROS) have been implicated in several models of neurodegeneration [16,17]. NADPH oxidase (NOX), an enzyme that produces ROS, is activated in brains from Alzheimer's disease (AD) [18] and Parkinson's disease (PD) [19]. NOX is a multi-subunit enzyme complex that is activated and induced by inflammatory signals [20,21]. The catalytic subunit of NOX, gp91^phox^, produces superoxide that can be toxic to neurons. In the present study, we find increased levels of NOX-gp91^phox^, and reactive oxygen species (ROS) following chronic ethanol treatment. Using transgenic mice that mark NF-κB transcription through induction of enhanced GFP mice (NF-κB^EGFP^) [22,23] we find NF-κB transcription, NOX gp91^phox ^activation and ROS production occur within the same cell. Further, increased levels of NOX-gp91^phox ^and cell death markers within orbital frontal cortex (OFC) are found in both chronic ethanol treated mouse and human post-mortem alcoholic brain. Our data indicate ethanol activation of NF-κB transcription of proinflammatory genes and formation of NOX-ROS play a pivotal role in ethanol induced neurodegeneration.

## Methods

### Animals

Eight-week male (20-22g) C57BL/6 mice were purchased from Jackson Laboratories (Bar Harbor, Maine). NF-κB enhanced GFP mice were gift from Dr. Christian Jobin's lab. All protocols in this study were approved by the Institutional Animal Care and Use Committee and were in accordance with the National Institute of Health regulations for the care and use of animals in research.

### Human tissue

Human post-mortem brain tissue was obtained from the New South Wales Tissue Resource Center in Australia [ethics committee approval number: X11-0107]. Paraffin sections of orbitofrontal cortex (OFC) were used in this study. The detailed patients' medical history is presented in Table 1. Human alcoholic patients averaged with lifetime consumption of over 500 Kg of ethanol were compared to moderate drinkers who averaged less than 1 drink per day with lifetime consumption of less than 100 Kg of ethanol. Alcoholic neurodegeneration is associated with chronic high levels of alcohol consumption, whereas, there is no neurodegeneration associated with moderate drinkers [24-26]. Only individuals with alcohol dependence not complicated by liver cirrhosis or nutritional deficiencies were included in this study. The main causes of death were cardiovascular disease for both groups. Complete life-style, medical histories and brain function as well as post-mortem interval (the time between death and the brain was removed from the body); causes of death and alcohol consumption are documented. Tobacco smoking history is also documented. Tobacco smoking is common in alcoholism and often confounds studies of alcoholism. Within our human subjects 6 of 8 controls and alcoholics had a history of smoking, although 3 controls were ex-smokers whereas all 6 smoking alcoholics continued to smoke. All psychiatric and alcohol use disorder diagnoses are confirmed using the Diagnostic Instrument for Brain Studies that is compliant with the Diagnostic Statistical Manual of Mental Disorders and has demonstrated reliability [27].

**Table 1:** Case characteristics of Subjects used for immunohistochemical analyses - Alcohol Consumption

Group	Patients	Age at Death	Sex	PMI	Clinical Cause of Death	Lifetime Ethanol (gm)	Lifetime Drinks	Drinks/Yr	Drinks/Day
Control	1	44	Male	50	Ischaemic heart disease	69000	4929	259	0.71

Control	2	46	Male	29	Acute myocardial infarction	17300	1236	73	0.20

Control	3	48	Male	24	Ischaemic heart disease	59000	4214	183	0.50

Control	4	50	Male	30	Coronary heart disease	5500	393	16	0.04

Control	5	50	Male	40	Haemopericardium	9000	643	26	0.07

Control	6	53	Male	16	Dilated cardiomyopathy	102000	7286	260	0.71

Control	7	60	Male	28	Ischaemic heart disease	0	0	0	0.00

Control	8	62	Male	46	Ischaemic heart disease	5000	357	10	0.03


Alcoholic	1	44	Male	15	Ischaemic heart disease	639000	45643	1902	5

Alcoholic	2	45	Male	7.5	Drowning	1271000	90786	3026	8

Alcoholic	3	49	Male	44	Ischaemic heart disease	1181000	84357	2556	7

Alcoholic	4	49	Male	16	Coronary artery thrombosis	1278000	91286	2608	7

Alcoholic	5	50	Male	17	Ischaemic heart disease	1958000	139857	4371	12

Alcoholic	6	51	Male	27	Gastro intestinal haemorrhage	1863000	133071	3914	11

Alcoholic	7	61	Male	59	Myocarditis	5811000	415071	9224	25

Alcoholic	8	61	Male	23.5	Atherosclerotic cardiovascular disease	3158000	225571	5127	14

Brains were collected by the New South Wales Tissue Resource Center brain donor program at the University of Sydney. Complete life-style, medical histories and brain function as well as post-mortem interval (PMI, the time between death and the brain was removed from the body), causes of death and alcohol drinking histories are documented. Among these patients controls averaged less than 1 drink per day whereas alcoholics averaged over 10 drinks per day. Alcoholic neurodegeneration is associated with chronic high levels of alcohol consumption, whereas, there is no neurodegeneration associated with moderate drinkers is related to lifetime alcohol consumption [24-26]. Tobacco smoking history is also documented. Tobacco smoking is commone in alcoholism and often confounds studies of alcoholism. Within our human subjects 6 of 8 controls and alcoholics had a history of smoking, although 3 controls were ex-smokers whereas all 6 smoking alcoholics continued to smoke. Only individuals with alcohol dependence not complicated by liver cirrhosis or nutritional deficiencies were included in this study. All psychiatric and alcohol use disorder diagnoses are confirmed using the Diagnostic Instrument for Brain Studies that is compliant with the Diagnostic Statistical Manual of Mental Disorders and has demonstrated reliability [27].

### Reagents

Cleaved caspase-3 (Asp 175) antibody was from Cell Signaling Technology (Danvers, MA). Fluoro-Jade B and mouse Neu-N antibody were from Chemicon international (Temecula, CA). Rabbit anti-Iba1 antibody was purchased from Wako Pure Chemical Industries, Ltd. (1-2Doshomachi 3-Chome Chuo-ku Osaka 540-8605, Japan). Monoclonal anti-mouse gp91^phox ^was from Transduction Laboratories (Lexington, KY). Rabbit polyclonal anti-gp91^phox ^IgG was purchased from Upstate cell signaling solutions (Temecula, CA). Goat polyclonal gp91^phox ^(C-15) antibody was purchased from Santa Cruz Biotechnology, Inc. (Santa Cruz, CA). Rabbit polyclonal microtubule associated protein 2 (MAP2) antibody was purchased from Abcam (Cambridge, MA). Polyclonal Rabbit anti-Glial Fibrillary Acidic Protein was from DakoCytomation (Glostrup, Denmark). Hydroethidine was from Invitrogen Molecular Probes (Eugene, OR). All other reagents came from Sigma Chemical Co. (St. Louis, MO).

### Drug treatments

Sixty male C57BL/6 mice were randomly assigned to water control group (30 mice) and ethanol group (30 mice). The mice were treated intragastrically with water (control) or ethanol (5 g/kg, i.g., 25% ethanol w/v), with volumes matched, daily for 10 days. The average blood alcohol concentration at 1 hour after the first ethanol treatment and the last ethanol treatment was 302 mg/dl ± 12 (w/v, n = 10) and 297 mg/dl ± 11 (w/v, n = 10), respectively. The blood ethanol level is high and considered to model binge drinking [28]. Twenty mice from each group were sacrificed at 24 hr after the last dose of ethanol for mRNA and histochemistry. Ten mice from each group were sacrificed at 1 week after the last dose of ethanol for NOX gp91^phox ^immunostaining. For diphenyleneiodonium (DPI) treatment, forty male C57BL/6 mice were randomly assigned to 4 groups: control, EtOH, DPI and EtOH plus DPI (10 mice per group). The mice in EtOH and EtOH plus DPI groups were treated intragastrically with ethanol (5 g/kg, i.g., 25% ethanol w/v) daily for 10 days. The mice in control and DPI groups were gavaged with water daily for 10 days. DPI (3 mg/kg, i.p.) was given to mice at 0.5 hr and 24 hr after the last dose of ethanol. In both water and ethanol groups, mice were injected with saline, with volumes and time matched. Mice were sacrificed 3 hr after the last dose of DPI. For NF-κB transcription study, twenty male NF-κB enhanced GFP mice, a transgenic mouse expressing the enhanced GFP under the transcriptional control of NF-κB *cis *elements (cis-NF-κB^EGFP^) [22,23], were treated intragastrically with water (control, 10 mice) or ethanol (5 g/kg, i.g., 25% ethanol w/v, 10 mice), daily for 10 days. The mice were sacrificed 24 hr after the last dose of ethanol. For ROS analysis, male C57BL/6 or NF-κB enhanced GFP mice (10 mice per group) were treated intragastrically with water (control) or ethanol (5 g/kg, i.g., 25% ethanol w/v), daily for 10 days. Mice were injected with dehydroethidium (10 mg/kg, i.p.) in 0.5% carboxymethyl cellulose at 23.5 hr after the last dose of ethanol. Brains were harvested 30 min later and frozen sections (15 μm) were examined for hydroethidine oxidation product, ethidium accumulation, by fluorescence microscopy. All experiments were repeated 2 to 3 times.

### Real-time PCR analysis

Total RNA was extracted from the brain samples of C57BL/6 mice treated with ethanol or water, and reverse transcribed as described previously [29]. The primer sequences used in this study were as follows: NF-κB p65 (essential modulator), 5'-GGC GGC ACG TTT TAC TCT TT-3' (forward) and 5'-CCG TCT CCA GGA GGT TAA TGC-3' (reverse); β-actin, 5'- GTA TGA CTC CAC TCA CGG CAA A-3' (forward) and 5'-GGT CTC GCT CCT GGA AGA TG-3' (reverse). The SYBR green PCR master mix (Applied Biosystems, Foster City, CA) was used for real-time PCR analysis. The relative differences in expression between groups were expressed using cycle time (Ct) values normalized with β-actin, and relative differences between control and treatment groups were calculated and expressed as relative increases setting control as 100%.

### Immunohistochemistry

Mouse brains were fixed with 4% paraformidehide in Phosphate Buffered Saline (PBS) and processed for immunostaining as described previously [29]. Human postmortem brains were processed to Paraffin sections for immunohistochemistry. Microglia were stained with rabbit anti-Iba1 antibody. Mouse NOX membrane subunit gp91^phox ^was immunostained with monoclonal anti-mouse gp91^phox ^or rabbit polyclonal anti-gp91^phox ^IgG. Human gp91^phox ^was immunostained with goat polyclonal gp91^phox ^antibody. Caspase-3 was immunostained with polyclonal anti-cleaved caspase-3 antibody. Neurons were stained with Neu-N or MAP2 antibody. Astrocytes were labeled with GFAP antibody. Immunolabeling was visualized by using nickel-enhanced 3,3'-diaminobenzidinne (DAB) or Alexa Fluor 488 (green) or 555 (red) or 633 (blue) dye.

### In situ visualization of O_2_^- ^and O_2_^- ^- derived oxidant production

*In situ *visualization of O_2_^- ^and O_2_^- ^- derived oxidant production was assessed by hydroethidine histochemistry [19,30]. Mice were injected with dehydroethidium (10 mg/kg, i.p.) in 0.5% carboxymethyl cellulose at 23.5 hrs after the last dose of ethanol. Brains were harvested 30 min later and frozen sections (15 μm) were examined for hydroethidine oxidation product, ethidium accumulation, by fluorescence microscopy (excitation 510 nm; emission 580 nm).

### Fluoro-Jade B staining with Neu-N labeling

Brain sections were immunostained with mouse Neu-N (a neuronal marker) antibody. Immunolabeling was visualized by using Alexa Fluor 555 dye. Sections were rinsed three times with PBS and one time with water before performing Fluoro-Jade B procedure. Sections stained with Neu-N were mounted on superfrost/plus microscope slides and air dried overnight. The sections were rinsed in distilled water for 2 min to rehydrate and transferred to a solution of 0.06% potassium permanganate for 10 min. The sections were then rinsed in distilled water for 2 min and placed in a 0.0004% Fluoro-Jade B solution made by adding 4 ml of a 0.01% stock solution of Fluoro-Jade B to 96 ml of 0.1% acetic acid. After 20 min in the Fluoro-Jade B staining solution, the stained slides were thoroughly washed in distilled water, dehydrated and coverslipped.

### Microscopic quantification

Immunoreactivity of mouse gp91^phox ^and fluorescent intensity of Fluoro-Jade B and ethidium were quantified using Bioquant Image Analysis Software (Nashville, TN). Images were captured on an Olympus BX51 microscope and Sony DCX-390 video camera at 40X. Light levels were normalized to preset levels and the microscope, camera, and software were background corrected to ensure reliability of image acquisition [31]. In each region (cortex and dentate gyrus), six random images from each brain sample were captured within a standard ROI (Region of Interest), the density of immunostaining and fluorescence was measured in pixels within this area (pixels/mm^2^). Subsequently, the average of the six measurements was used to represent the immunoreactivity or fluorescence intensity of each sample. When measuring fluorescence intensity in the cells, we eliminated the background by adjusting threshold to avoid background staining. For +IR cells counting, a modified stereological method was used to quantify cells within regions of interest following immunostaining of brain sections using the CAST stereological system [32,33]. Specifically, cell density (**N_v_) **of caspase-3 and gp91^phox ^+ immunoreactivity (+IR) was determined following the optical disector method [34,35], which was calculated as follows:

$${\text{N}}_{\text{v}}=\frac{\sum \text{Q}}{\sum \text{disector}\times \text{A}\left(\text{fr}\right)\times \text{h}}$$

Where ∑Q is the sum of the caspase-3 or gp91phox + IR cells counted from each disector frame, ∑disector is the sum of the number of disector frames counted, A(fr) is the known area associated with each disector frame, and h is the known distance between two disector planes (we used 10 μm).

For colabeling study, double or triple stained sections were digitally photographed with Leica SP2-AOBS confocal microscope and analyzed with Leica SP2 LCS software.

### Statistical analysis

The data are expressed as mean ± SEM and statistical significance was assessed with an ANOVA followed by Bonferroni's t-test using the StatView program (Abacus Concepts, Berkeley, CA). A value of P < 0.05 was considered statistically significant.

## Results

### Chronic ethanol increases caspase-3 expression and Fluoro-Jade B staining

**Figure 1:**
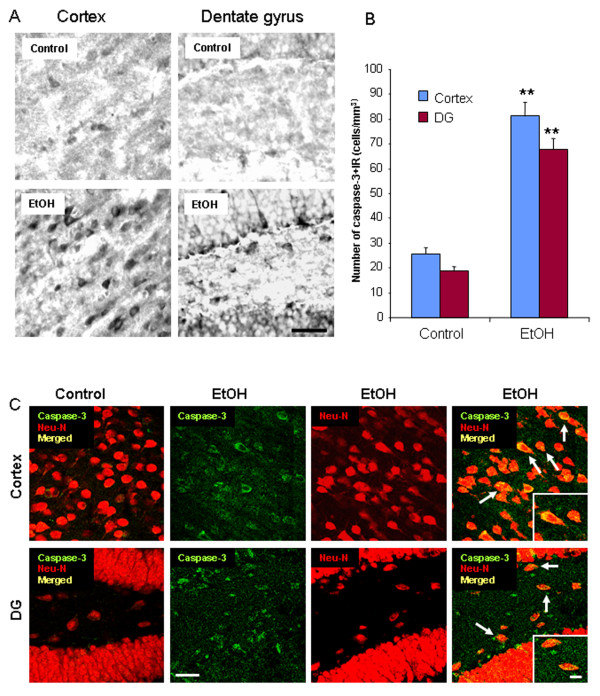
**Chronic ethanol increases caspase-3 activation**. (A) Representative images of activated caspase-3+immunoreactive (+IR) cells in cortex and dentate gyrus (DG) from control and ethanol-treated mice. (B) Quantification of caspase-3 immunoreactivity in cortex and DG. Ethanol-treated mouse brains showed more caspase-3+IR cells than water controls 24 hrs after the last dose of ethanol treatment. (C) Brain sections from water and ethanol (5 g/kg, i.g., 10 days) treated C57BL/6 mice were double-stained with cleaved caspase-3 and Neu-N (a neuronal marker) antibodies. Shown are images of cortex and DG for activated caspase-3 (green), Neu-N (red), and colabeling of caspase-3 and Neu-N (yellow). Confocal microscopy shows that caspase-3 is expressed in Neu-N+IR cells, as shown with arrows indicating the colabeling of caspase-3 and Neu-N in ethanol-treated mice. Control sections show little or not colabeling of caspace-3 and Neu-N+IR cells. Inset is the higher magnification of arrow pointed cells. ** P < 0.01, compared with the water control mice (n = 10). Scale bar = 30 μm; inset 5 μm.

**Figure 2:**
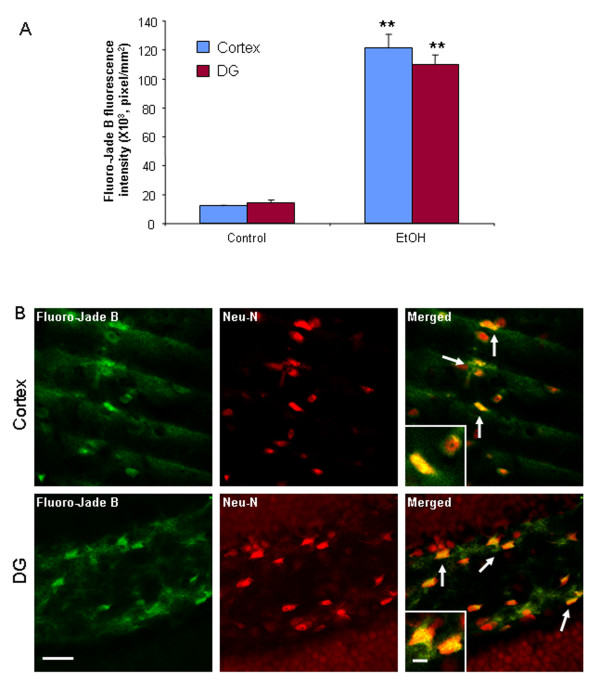
**Fluoro-Jade B staining in the cortex and dentate gyrus of C57BL/6 mice**. Brain sections from C57BL/6 mice treated with either water or ethanol daily for 10 days were double-stained with Neu-N antibody and Fluoro-Jade B as described in Materials and Methods. Neu-N immunolabeling was visualized by using Alexa Fluor 555. (A) Fluorescent intensity of Fluoro-Jade B positive cells in cortex and dentate gyrus was quantified by BioQuant image analysis system. The cortex and DG of chronic ethanol-treated mice had more Fluoro-Jade B positive cells than water control mice 24 hrs after the last dose of ethanol treatment. These cells are colocalized with Neu-N, a neuronal marker, suggesting neuronal death. (B) Representative images for Fluoro-Jade B (green), Neu-N (red) and colabeling of Fluoro-Jade B and Neu-N (yellow) from cortex and dentate gyrus in ethanol-treated mice. ** P < 0.01, compared with the water control mice (n = 10). Scale bar = 30 μm; inset 5 μm.

**Figure 3:**
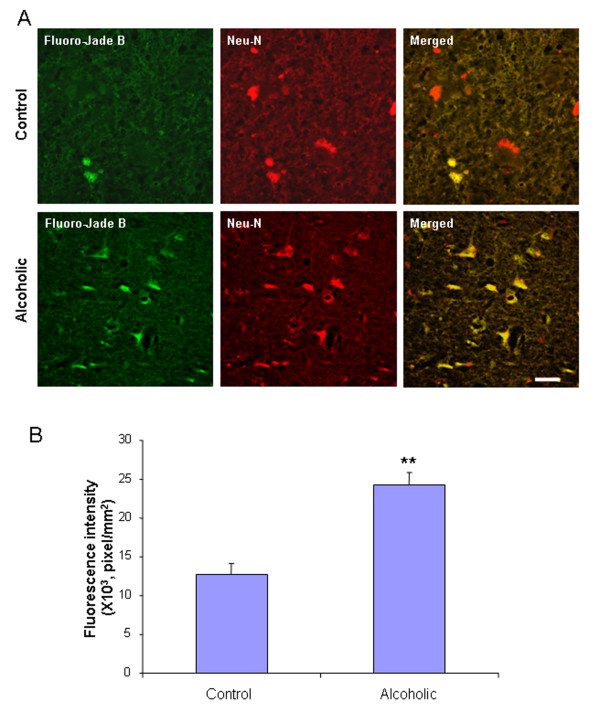
**Increased Fluoro-Jade B staining in human post-mortem alcoholic brain (A) Representative images from the orbitofrontal cortex (OFC) of moderate drinking control brain and alcoholic brain stained with Fluoro-Jade B**. Brain sections from the OFC of human post-mortem alcoholics and moderate drinkers were double-labeled with Neu-N antibody and Fluoro-Jade B. Confocal microscopy indicated that Fluoro-Jade B positive cells were colocalized with Neu-N, suggesting neuronal death Scale bar = 30 μm. (B) Level of fluorescent intensity of Fluoro-Jade B positive cells was quantified by BioQuant image analysis software. The OFC of human post-mortem alcoholic brain had significant increase in fluorescent intensity of Fluoro-Jade B positive cells, compared to the OFC of human moderate drinking control brain (** P < 0.01, n = 8).

To determine the effect of ethanol exposure on neurodegeneration in mice, immunohistochemistry for cleaved caspase-3 [36] and Fluoro-Jade B histochemistry were performed on C57BL/6 mouse brain sections treated with water or ethanol (5 g/kg, i.g.) daily for 10 days. Ethanol-treated mice showed an increase in activated caspase-3 immunoreactivity 24 hours after the last dose of ethanol treatment, compared to water controls (Figure 1A). The number of activated caspase-3+immunoreactive (IR) cells increased 3.1 fold in cortex and 3.5 fold in dentate gyrus in ethanol-treated mice (Figure 1B). To determine if caspase-3+immunoreactivity (+IR) was neuronal, double immunohistochemistry for cleaved caspase-3 and Neu-N, a neuronal marker was used. Confocal microscopy indicated that most activated caspase-3 +IR cells colocalize with Neu-N+IR cells (Figure 1C), suggesting chronic ethanol exposure causes neuronal cell death. Fluoro-Jade B, another cell death marker, was also used to assess ethanol-induced neurotoxicity [37]. Brain sections from control animals showed little or no Fluoro-Jade B staining. However, mouse brains exposed to chronic ethanol increased 10 fold in cortex and 7.6 fold in dentate gyrus in intensity of Fluoro-Jade B positive staining 24 hours after the last dose of ethanol treatment compared to water control group (Figure 2A). Confocal microscopy indicated that most Fluoro-Jade B positive cells were colocalized with Neu-N+IR (Figure 2B). Both cleaved caspase-3+IR and Fluoro-Jade B markers suggest chronic ethanol induce neuronal cell death in C57BL/6 adult mice. The orbitofrontal cortex (OFC) of human post-mortem brain was assessed using Fluoro-Jade B. The OFC of human moderate drinking control brain showed few Fluoro-Jade B cells whereas the OFC of alcoholic brain showed more labeled cells (Figure 3). Confocal microscopy found that Fluoro-Jade B positive cells in human brain were mostly colocalized with Neu-N, suggesting increased neuronal cell death in human post-mortem alcoholic brain.

### Chronic ethanol induces activation of microglia and astrocytes

**Figure 4:**
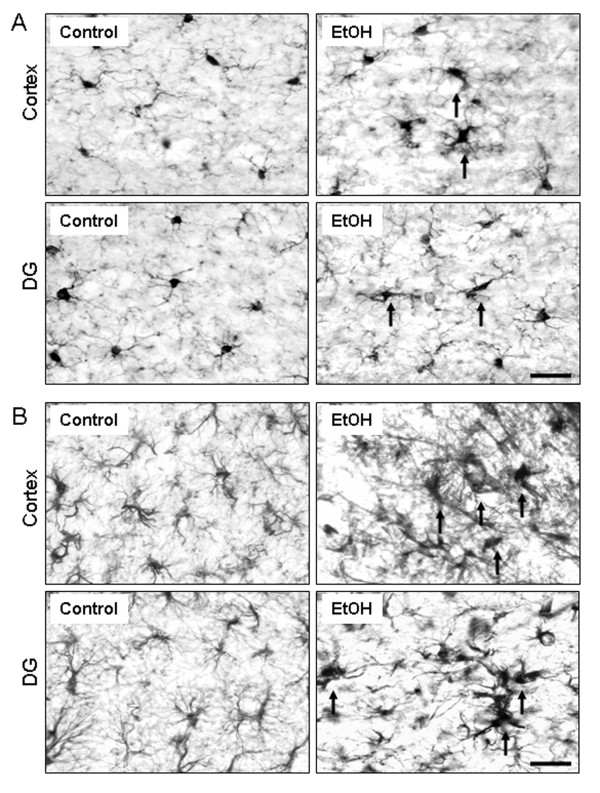
**Immunohistochemical analysis of microglia and astraglia**. Male C57BL/6 mice were treated intragastrically with water or ethanol (5 g/kg, i.g.) daily for 10 days and sacrificed 24 hours following ethanol treatment. Brain sections were stained with Iba1 microglial antibody or GFAP astroglial antibody. (A) Chronic ethanol exposure induced microglial activation in many brain regions. Images shown are representative of Iba1+IR cells in cortex and dentate gyrus from control and ethanol groups. In water control mice, most of the microglia were in a resting morphological shape. However, Iba1+IR cells in ethanol-treated mouse brains were shown by increased cell size, irregular shape, and intensified Iba1 staining consistent with morphological changes in activated microglia. Scale bar = 200 μm. (B) Images are representative of GFAP+IR cells in cortex and dentate gyrus from water and ethanol groups. Chronic ethanol markedly increased astroglial activation: upregulation of GFAP immunoreactivity and morphology of hypertophic astrocytes. Scale bar = 200 μm.

Previous studies have linked activation of microglia, production of proinflammatory factors and reactive oxygen species (ROS) to neurodegeneration [16,29,38]. Our previous research found that 10 daily doses of ethanol significantly increased levels of brain proinflammatory genes (TNFα and MCP-1, etc.) [9]. To investigate proinflammatory responses in this experiment sections were immunostained with Iba1 microglial antibody. In the water control group, microglia have a resting morphology. Ethanol treated mouse brains showed activated microglia morphology in multiple brain regions, including cortex and dentate gyrus of hippocampus (Figure 4A) 24 h after the last dose of ethanol. Microglia activation following ethanol treatment is indicated by increased cell size, irregular shape, intensified Iba1 staining, and an altered ameboid morphology. Thus, Iba1+ IR morphological assessment indicate ethanol causes microglial activation.

Astrocyte activation was assessed by morphology using GFAP, an astrocyte-specific intermediate filament protein [39,40]. Chronic ethanol treatment increased GFAP + IR in cortex and dentate gyrus (Figure 4B) 24 h after the last dose of ethanol. In addition to these two brain regions, astroglial activation was also notably observed in other brain areas, such as substantia nigra and forceps minor corpus callosum in the ethanol treated-mice (data not shown). Thus, the data together with increased cell death markers (caspase-3 and Fluoro-Jade B) by chronic ethanol treatment suggest that astroglial activation mediate ethanol-induced neurodegeneration.

### Chronic ethanol enhances NF-κB mRNA and protein expression

**Figure 5:**
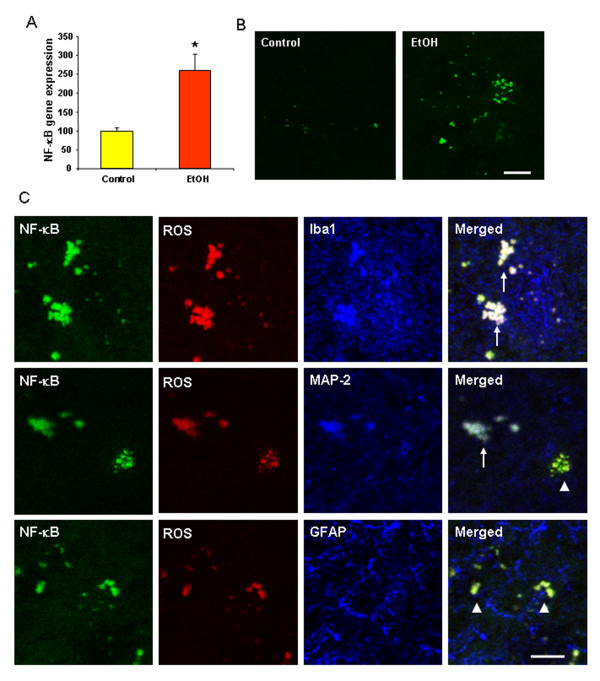
**Chronic ethanol enhances NF-κB mRNA and protein expression**. C57BL/6 and NF-κB enhanced GFP mice were treated with water or ethanol (5 g/kg, i.g.) daily for 10 days and sacrificed 24 hrs after the last dose of ethanol. (A) C57BL/6 mouse brain mRNA expression of NF-κB-p65 is significantly increased by 10 doses of ethanol exposure 24 hrs following ethanol treatment compared with water controls, as determined by real-time PCR. (B) Representative images are from the dentate gyrus of NF-κB enhanced GFP mice 24 hrs after water or ethanol treatment. Ethanol treated mouse brain had more NF-κB-GFP (green fluorescence) than water control brain. Scale bar = 20 μm. (C) NF-κB reporter mice were injected with dehydroethidium (10 mg/kg, i.p.) 23.5 hrs after ethanol treatment. Brains were harvested 30 min later and frozen sections (15 μm) were processed and immunostained for Iba1, Neu-N or GFAP to analyze cell phenotype for NF-κB activation and ROS production. Confocal results reveal that NF-κB activation and ROS production predominantly occurred in microglia and neurons as shown by NF-κB (green) and ROS (red) are triple labeled with Iba-1+IR cells (blue) or with MAP-2 +IR neurons (blue). Triple-labeling is shown in write with arrows. Double-labeled images are shown in yellow with arrowheads indicating the colabeling of NF-κB (green) and ROS (red). The images presented are from dentate gyrus of ethanol treated mice. Scale bar = 10 μm. * P < 0.05, compared with the water control mice.

Previous studies have suggested ethanol activates nuclear factor κB (NF-κB) transcription inducing expression of proinflammatory genes [14,41]. To investigate effect of ethanol on NF-κB mRNA and protein expression, C57BL/6 and NF-κB-GFP reporter mice were treated intragastrically with water (control) or ethanol (5 g/kg, i.g., 25% ethanol w/v) daily for 10 days as before and sacrificed 24 hrs after the last dose of ethanol. Chronic ethanol significantly increased NF-κB-p65 gene expression in C57BL/6 mouse brain (Figure 5A). In NF-κB GFP reporter mice ethanol treatment markedly increased GFP Fluorescence in multiple brain regions, such as dentate gyrus (Figure 5B).

Cell phenotype for NF-κB activation and ROS production was examined using histochemical markers. NF-κB enhanced GFP reporter mice showed green fluorescence. ROS were detected by red hydroethidine histochemistry and cell type markers, e.g. Iba1 microglial antibody, MAP2 neuronal antibody and GFAP astroglial antibody. Confocal microscopy found that NF-κB and ROS were triple-labeled with Iba1 or MAP2, but not with GFAP (Figure 5C), indicating ethanol-induced NF-κB activation and ROS production mostly occurred in microglia and neurons.

### Chronic ethanol increases expression of NOX and production of ROS

**Figure 6:**
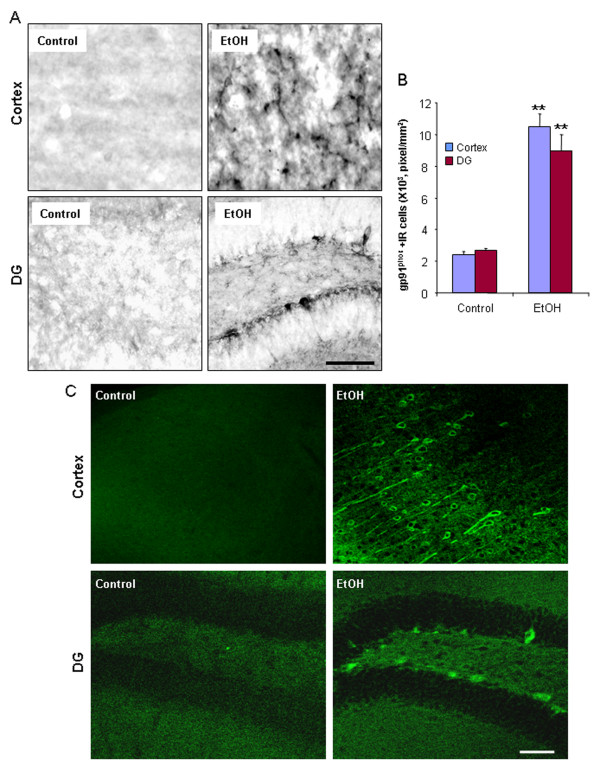
**Chronic ethanol up-regulates brain NOX subunit gp91^phox ^expression**. Brain sections from C57BL/6 mice treated with water or ethanol (5 g/kg, i.g.) daily for 10 days and sacrificed 24 hrs and 1 week after ethanol exposure were stained with monoclonal mouse gp91^phox ^antibody. Immunolabeling was visualized by using nickel-enhanced 3,3'-diaminobenzidinne (DAB) for 24 h samples and Alexa Fluor 488 dye (green) for 1 week samples. (A) The images shown are representative of gp91^phox ^+IR cells in cortex and DG 24 hrs after water and ethanol exposure. Scale bar = 200 μm. (B) Chronic ethanol significantly increased the level of gp91^phox ^immunoreactivity 24 hrs after ethanol treatment, as quantified by BioQuant image analysis system. (C) Chronic ethanol-induced increase in gp91^phox ^expression remained elevated at 1 week after ethanol treatment. Scale bar = 100 μm. ** P < 0.01, compared with the water control mice.

NADPH oxidase (NOX) is the enzyme complex responsible for the respiratory burst in phagocytes. Activation of this enzyme in microglia causes the production of ROS leading to dopaminergic neurotoxicity [29]. To determine whether NOX is involved in chronic ethanol induced neurotoxicity we investigated the expression of NOX gp91^phox^, the catalytic subunit of phagocytic oxidase commonly associated with proinflammatory responses. Cortex and dentate gyrus of ethanol treated mouse brain had significantly more gp91^phox ^+IR cells, compared to those of water control brain 24 hrs after ethanol treatment (Figure 6A). Quantification of gp91^phox ^+IR indicated that ethanol induced gp91^phox ^+IR 4.3 fold in cortex and 3.4 fold in DG (Figure 6B). Chronic ethanol-induced increases in gp91^phox ^expression remained elevated 1 week after ethanol treatment (Figure 6C). Thus, ethanol treatment of mice increases NOX gp91^phox ^expression that persists long after cessation of drinking.

**Figure 7:**
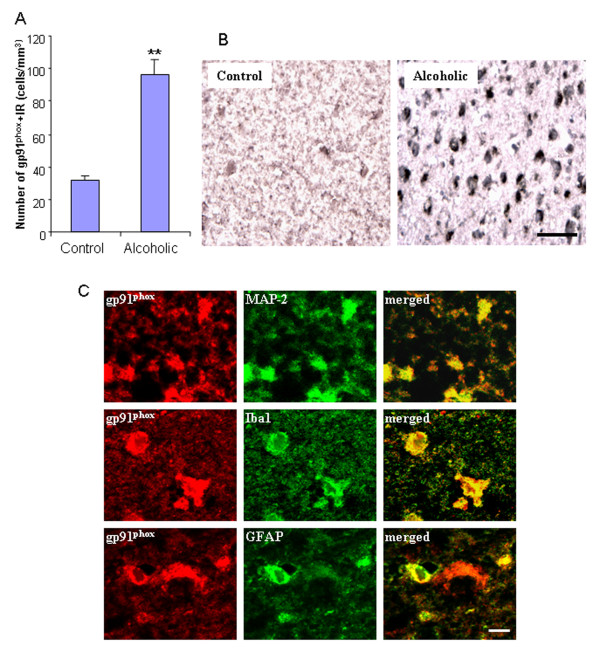
**Increased NOX subunit gp91^phox ^expression in human alcoholic brain**. (A) Quantification of gp91^phox ^+ IR cells. The orbitofrontal cortex (OFC) of human postmortem alcoholic brain has a significant increase in the number of gp91^phox ^+ IR cells, compared to the OFC of human moderate drinking control brain (** P < 0.01, n = 8). (B) The representative images from the OFC of moderate drinking control brain and alcoholic brain stained with gp91^phox ^antibody. Scale bar = 50 μm. (C) Colocalization of NOX subunit gp91^phox ^. Brain sections from the OFC of human alcoholic brains were double-labeled for gp91^phox ^(red) and MAP-2 (a neuronal marker), Iba1 (a microglial marker), and GFAP (an astroglial marker) (green) to analyze colabeling by using the Leica SP2 LCS confocal software. Confocal microscopy shows that gp91^phox ^+IR cells are colocalized with MAP-2, Iba-1, and GFAP+IR cells as shown in yellow, indicating NOX subunit gp91^phox ^is expressed in neurons (the upper panel), microglia (the middle panel) and astroglia (the lower panel). Scale bar = 10 μm. ** P < 0.01, compared with the OFC of human moderate drinking control brain.

Human postmortem alcoholic brain histochemistry showed significant increases in the number of gp91^phox ^+ IR cells, compared to human moderate drinking control brain (Figure 7A, B). Double antibody studies with cell specific markers and confocal microscopy reveal that gp91^phox ^+IR cells in human postmortem alcoholic brain are colocalized with a neuronal marker (MAP-2), a microglial marker (Iba-1), and an astroglial marker (GFAP) (Figure 7C). These results indicate that alcohol increases NOX gp91^phox ^expression in both ethanol-treated mouse brain and human alcoholics consuming large amounts of alcohol.

**Figure 8:**
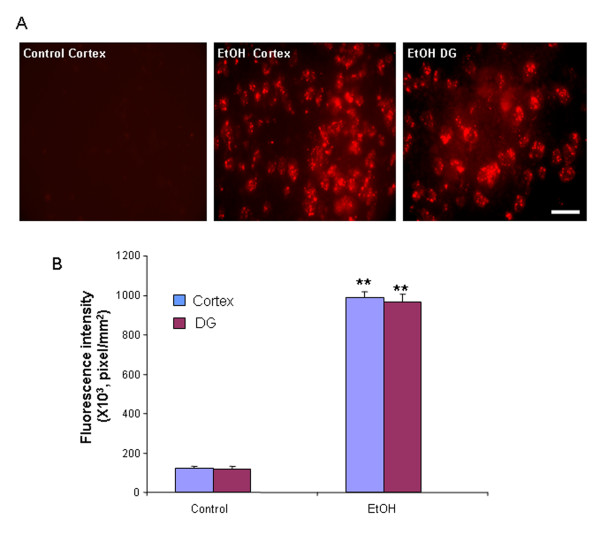
**Chronic ethanol significantly increased O_2_^- ^and O_2_^- ^-derived oxidant production**. Male C57BL/6 mice were treated with water or ethanol (5 g/kg, i.g.) daily for 10 days and injected with dehydroethidium (10 mg/kg, i.p.) 23.5 hrs after the last dose of ethanol exposure. Brains were harvested 30 min later and frozen sections (15 μm) were examined for hydroethidine oxidation product, ethidium accumulation, by fluorescent microscopy. (A) Representative images of ethidium fluorescence in cortex and dentate gyrus. In the cortex and DG of control mouse brain, ethidium fluorescence was minimal (DG image not shown). Both cortex and DG of ethanol treated mice had significant increases in ethidium fluorescence. Scale bar = 200 μm. (B) Level of fluorescent intensity of ethidium positive cells was quantified by BioQuant image analysis software. Ethanol treatment of C57BL/6 mice showed significantly increased O_2_^- ^and O_2_^-^-derived oxidant production in either cortex or DG, compared with water control mice. ** P < 0.01, compared with water controls.

To investigate whether induced NOX produces superoxide in brain, we used *in situ *visualization of reactive oxygen species, e.g. O_2_^- ^and O_2_^-^-derived oxidant production of ethidium from hydroethidine *in vivo *[19,30]. In vehicle-treated mice, there was little to no detection of O_2_^- ^and O_2_^-^-derived oxidant production of ethidium. In ethanol-treated mice, a significant production of O_2_^- ^and O_2_^-^-derived oxidants was observed 24 hrs after 10 daily doses of ethanol exposure (Figure 8A). Quantification of the ethidium fluorescence indicates that ethanol increased O_2_^- ^and O_2_^-^-derived oxidants more than 7 fold in cortex and DG, compared to controls (Figure 8B). These findings indicate that ethanol increases expression of NOX gp91^phox ^and increases the formation of reactive oxygen species. Human alcoholics show a similar increase in NOX+IR consistent with chronic alcohol abuse in humans increasing proinflammatory oxidative stress in brain.

### The cellular localization of chronic ethanol-induced activation of NOX and production of ROS

**Figure 9:**
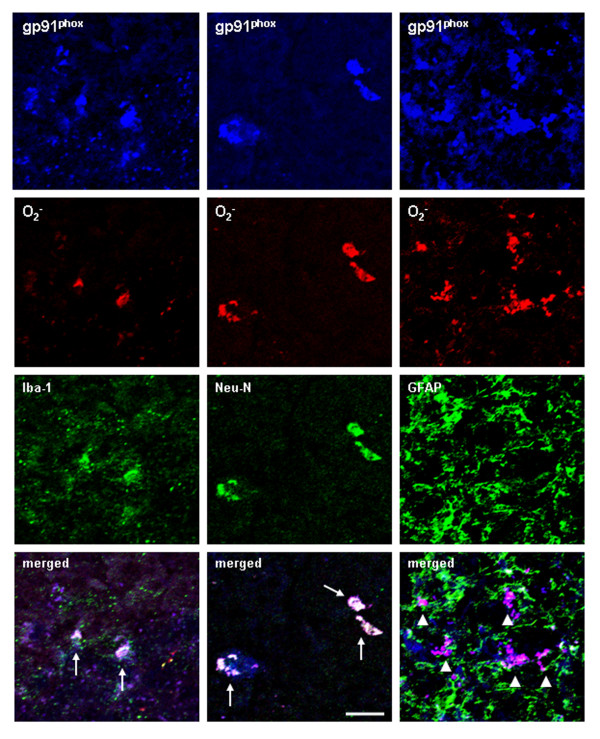
**Determination of cell phenotype for NOX subunit gp91^phox ^expression and O_2_^- ^production**. Cell phenotype was determined by confocal microscopy of sections triple labeled for gp91^phox ^(blue), O_2_^- ^(red) with Iba-1, or Neu-N or GFAP (green). C57BL/6 mice were injected with dehydroethidium (10 mg/kg, i.p.) 23.5 hrs after ethanol treatment. Brains were harvested 30 min later and frozen sections (15 μm) were processed and double-immunostained for gp91^phox ^and Iba1, gp91^phox ^and Neu-N as well as gp91^phox ^and GFAP to analyze triple labeling or colabeling by using the Leica SP2 LCS confocal software. Confocal microscopy shows that gp91^phox ^+IR cells are triple-labeled with O_2_^- ^and Iba1 or Neu-N, but not with GFAP. Triple-labeled representative images are shown in white with arrows indicating the triple-labeling of gp91^phox ^with O_2_^- ^and Iba1 (merged, the lower left panel) or gp91^phox ^with O_2_^- ^and Neu-N (merged, the lower middle panel). Double-labeled representative images are shown in pink with arrowheads indicating the colabeling of gp91^phox ^with O_2_^- ^, but not with GFAP (merged, the lower right panel). The images presented are from dentate gyrus of ethanol treated mice. Scale bar = 20 μm.

To determine if ROS are found in cells expressing NOX gp91^phox^, we performed triple labeling using gp91^phox^+IR together with antibodies against the Iba1, a marker of microglia or Neu-N, a marker of neurons, or GFAP, a marker of astrocytes on brain sections from the mice injected with dehydroethidium, an agent detecting ROS. Confocal microscopy indicated that gp91^phox ^(blue) and O_2_^- ^(red) labeling was not detectable in water control mouse brains (picture not shown). However, intense fluorescence of gp91^phox ^and O_2_^- ^was observed in ethanol treated mouse brains (Figure 9, first and second panels) 24 h after the last dose of ethanol. Triple-labeled cells are white due to gp91 (blue), O_2 _(red) combining with microglial (Iba1, green) or neuronal (Neu-N, green) marker proteins (Figure 9), but not with astrocyte GFAP (pink). These results indicate that chronic ethanol-induced activation of NOX and production of O_2_^- ^predominantly occurs in microglia and neurons in mouse brain.

### DPI reduces chronic ethanol-induced microglial activation and ROS generation

**Figure 10:**
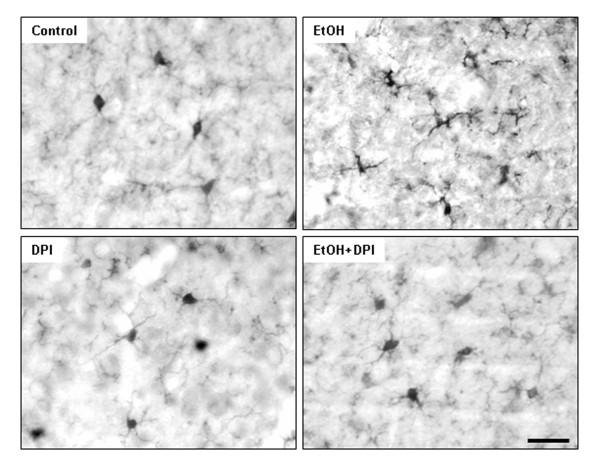
**Diphenyleneiodonium (DPI) inhibited ethanol-induced microglial activation**. Male C57BL/6 mice were treated intragastrically with water or ethanol (5 g/kg, i.g.) daily for 10 days. Diphenyleneiodonium (DPI) (3 mg/kg) was injected intraperitoneally 0.5 hr and 24 hr after the last dose of ethanol. Mice were sacrificed 3 hrs after the last dose of DPI. Brain sections were stained with Iba-1 antibody. In water control mice, most of the microglia were in a resting morphological shape. Iba1+IR cells in ethanol-treated mouse brains were activated as shown by increased cell size, irregular shape, and intensified Iba1 staining. DPI inhibited ethanol-induced microglial activation shown in a resting morphological shape. Scale bar = 200 μm.

**Figure 11:**
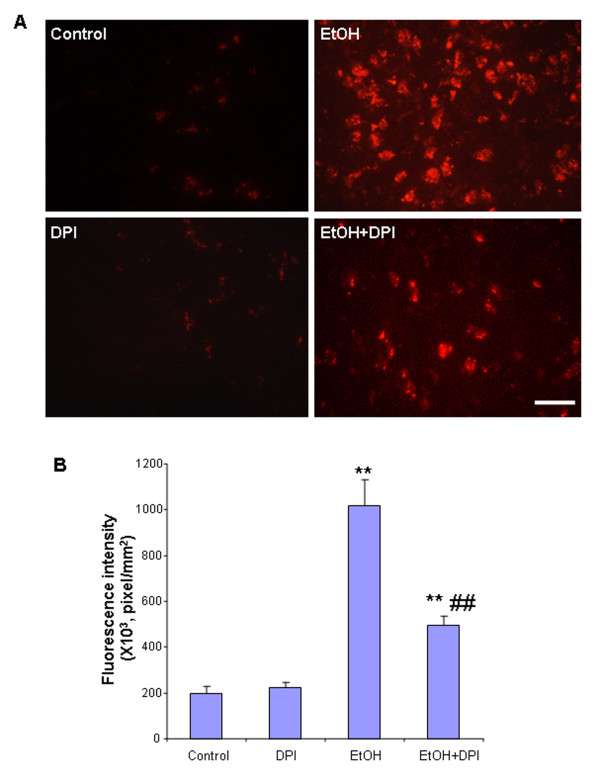
**DPI inhibited ethanol-induced production of O_2_^- ^and O_2_^- ^-derived oxidants**. C57BL/6 mice treated with water, ethanol, DPI and ethanol plus DPI were injected with dehydroethidium (10 mg/kg, i.p.) 2.5 hrs after the last dose of DPI. Brains were harvested 30 min later and frozen sections (15 μm) were examined for hydroethidine oxidation product, ethidium accumulation, by fluorescent microscopy. (A) The images demonstrated that ethanol markedly increased production of O_2_^- ^and O_2_^-^-derived oxidants compared to water controls, and DPI significantly reduced ethanol-induced production of O_2_^- ^and O_2_^-^-derived oxidants compared to ethanol-treated group. Scale bar = 200 μm. (B) Level of fluorescent intensity of ethidium positive cells in cortex was quantified by BioQuant image analysis software. Ethanol treatment of C57BL/6 mice showed significantly increased O_2_^- ^and O_2_^-^-derived oxidant production, compared with water control mice. DPI significantly decreased ethanol-induced increases in the fluorescent intensity of O_2_^- ^and O_2_^-^-derived oxidant production. ** P < 0.01, compared with water controls. ## P < 0.01, compared with ethanol-treated group.

In an effort to discern the role of ROS in ethanol-induced neurotoxicity, we used a NOX inhibitor, Diphenyliodonium (DPI) [42,43]. As the resident innate immune cells in the brain, microglia are a predominant source of pro-inflammatory factors [TNFα, MCP-1, IFN-γ and oxidative stress (NO, H_2_O_2_, O_2_^- ^and OHOO^-^/ONOOH)], which are toxic to neurons [44]. To examine whether ethanol-induced microlgial activation is associated with ROS and the consequent neurotoxicity, C57BL/6 mice were injected with DPI. As shown in Figure 10, after 10 daily doses of ethanol treatment, microglia appear activated: increased cell size, irregular shape, and intensified Iba1 staining. Treatment with DPI significantly reduced microglial activation by ethanol exposure. In DPI and ethanol co-treatment group, microglia showed the resting morphology similar to those in the water control group. These results suggest an important role of NOX in ethanol-induced microglia activation. To analyze the effects of DPI treatment on ROS, hydroethidine histochemistry was performed. Exposure of mice to ethanol for 10 days was found to significantly increase ROS generation (Figure 11A, B), again providing confirmation that ethanol can elicit ROS generation in adult C57BL/6 mouse brain. DPI treatment caused 51% decrease in fluorescence intensity of O_2_^- ^and O_2_^-^-derived oxidants, compared with ethanol-treated group (Figure 11B), suggesting that NOX-generated ROS contribute to chronic ethanol-induced microglial activation.

### Inhibition of NOX with DPI prevents ethanol-induced neurodegeneration

**Figure 12:**
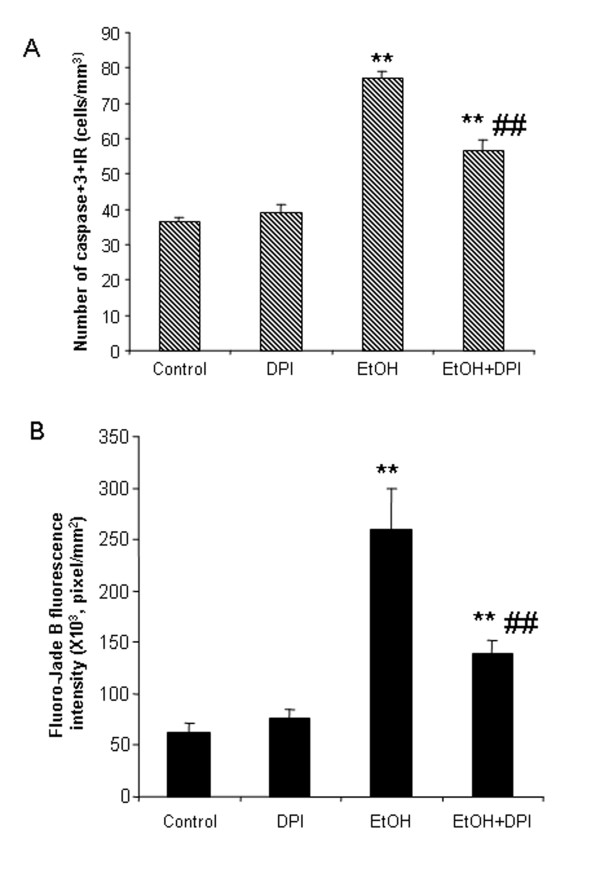
**DPI reduced ethanol-induced caspase-3 activation and Fluoro-Jade B staining**. Male C57BL/6 mice were treated intragastrically with ethanol (5 g/kg, i.g.) daily for 10 days. DPI (3 mg/kg) was injected intraperitoneally 0.5 hr and 24 hr after the last dose of ethanol. Mice were sacrificed 3 hrs after the last dose of DPI. Brain sections were stained with polyclonal cleave caspase-3 antibody and Fluoro-Jade B, respectively. (A) Ethanol increased the number of caspase-3+IR cells. DPI significantly reduced ethanol-induced increases in the number of caspase-3+IR cells. (B) Fluorescent intensity of Fluoro-Jade B positive cells in cortex was quantified by BioQuant image analysis system. Ethanol-treated mouse brains showed more Fluoro-Jade B positive cells than water-treated mouse brains. DPI significantly decreased ethanol-induced increases in the fluorescent intensity of Fluoro-Jade B positive cells. ** P < 0.01, compared with water controls. ## P < 0.01, compared with ethanol-treated group.

Chronic ethanol increased the number of activated caspase-3+IR cells by 104% compared to water control group. DPI alone did not show any effect on caspase-3 immunolabeling compared to water controls. Co-treatment with DPI and ethanol reduced ethanol-induced increase in caspase-3+IR cells (Figure 12A). Also, DPI significantly reduced ethanol increased Fluoro-Jade B fluorescence intensity by about half (Figure 12B). DPI is a NOX inhibitor. DPI treatment reduced ROS and cell death markers (caspase-3 and Fluoro-Jade B), so the data suggest that NOX-generated ROS contribute to chronic ethanol-induced neurotoxicity.

## Discussion

Chronic binge drinking can cause brain damage, cognitive dysfunction and neurodegeneration. Cerebral white matter atrophy and neuronal loss in the frontal cortex, the hypothalamus, and the thalamus are found in alcoholic brains [1,45]. Binge ethanol treatment of adult rats induces neuronal damage [8]. We have recently discovered that alcohol increases proinflammatory cytokines (TNFα, IL-1β), chemokine (MCP-1) and microglial activation in mouse brain that mimic increases found in post-mortem human alcoholic brain [9,10]. Here, our data, for the first time, find that 10 daily binge doses of ethanol caused significant increases in the staining of cell death markers: cleaved caspase-3 and Fluoro-Jade B. Activated caspase-3+immunoreactivity (+IR) is a putative marker for dying cells [36]. Fluoro-Jade B is an alternative marker selectively staining degenerating neurons in the central nervous system (CNS) [37]. Our data found that chronic ethanol increased the number of activated caspase-3+IR cells 3.1 fold in cortex and 3.5 fold in dentate gyrus (Figure 1). Fluoro-Jade B positive cells was increased 10 fold in cortex and 7.6 fold in dentate gyrus (Figure 2). These results suggest that chronic ethanol can cause neurodegeneration in adult mice. We also studied human post-mortem alcoholic frontal cortex, the brain region most associated with alcoholic neurodegeneration [46-48]. We found that the orbitofrontal cortex (OFC) of human postmortem alcoholic brain has significantly more Fluoro-Jade B positive cells which are colocalized with Neu-N, a neuronal marker, compared to the OFC of human moderate drinking control brain. Together, these results indicate that alcohol can cause neurodegeneration in adult mice that mimics that found in human alcoholics.

The underlying mechanism of alcohol-induced brain damage is not well understood. Activation of glial cells is a critical event in many neuroinflammatory processes [49,50]. Activation of microglia has been linked to neurodegeneration through the production of neurotoxic factors, such as proinflammatory cytokines and free radicals [51,52]. Here we show that 10 doses of ethanol-treated mouse brain displayed the characteristics of activation of microglia: increased cell size, irregular shape, and intensified Iba-1 immunoreactivity (Figure 4A). We previously reported that chronic ethanol can activate microglia increasing proinflammatory factors (TNFα, IL-1β and MCP-1, etc.) [9]. Astroglial activation we report here is also observed 24 hours after chronic ethanol treatment (Figure 4B). The activated astroglia were shown by a marked upregulation of GFAP immunoreactivity along with hypertrophic astrocytes in several brain regions, including cortex and dentate gyrus. These results are consistent with Guerri lab's findings that show hypertrophic astrocytes as well as increased caspase-3+IR cells in the mice treated with chronic ethanol administration (10% ethanol, v/v, for 5 months) [11]. Reactive hypertrophic astrogliosis is a marker of neuroinflammation. Again, our data support that activation of microglia and astroglia contribute to chronic ethanol-induced neuroinflammation and neurodegeneration.

NF-κB is a family of transcription factors involved in regulating cell death/survival, differentiation, and inflammation. Acute ethanol administration has been demonstrated to activate the NF-κB system in the brain, and this in turn triggers the expression of TNFα as well as other proinflammatory cytokines and NF-κB-regulated genes [13]. Increases in NF-κB DNA-binding activity during ethanol treatment correlate with the increased expression of proinflammatory genes in hippocampal-entorhinal cortex slice cultures [14]. Blockade of NF-κB activation by p65 siRNA or the antioxidant butylated hydroxytoluene (BHT) reduces the induction of proinflammatory TNFα, IL-1β, MCP-1, protease TACE, tissue plasminogen activator (tPA) and inducible nitric oxide synthase by ethanol [14].

In rats BHT blocked NF-κB-DNA binding and ethanol neurotoxicity [13]. In this study, we find that 10 doses of ethanol significantly increase NF-κB -p65 gene expression (Figure 5A) in C57BL/6 mice. Consistent with the mRNA data, in ethanol treated group, NF-κB GFP reporter fluorescence was markedly increased in multiple brain regions, such as dentate gyrus in NF-κB enhanced GFP mice (Figure 5B). Increases occurred predominantly in microglia and neurons. There data support the hypothesis that ethanol-induced oxidative stress involves a neuroinflammatory mechanism under the regulation of NF-κB transcription.

Another novel discovery is that for the first time we show alcohol increases NADPH oxidase gp91^phox ^(NOX2) in adult mouse brain that mimics that found in human post-mortem alcoholic brain. NOX gp91^phox ^remained elevated 1 week after chronic ethanol treatment (Figure 6C). The orbitofrontal cortex (OFC) of human post-mortem alcoholic brain also had significant increases in the number of gp91^phox ^+ IR cells, compared to the OFC of human moderate drinking control brain (Figure 7A, B). Confocal microscopy of double IHC with markers specific for neurons, microglia and astrocytes indicated that human NOX gp91^phox ^was expressed in all 3 cell types in alcoholics (Figure 7C). Previous studies have found increased NOX-proinflammatory responses in mice can persist for at least 10 months and longer [38]. The persistence of NOX-proinflammatory responses suggests the elevated levels in human alcoholic brain may represent both recent alcohol drinking as well as heavy drinking periods earlier in the lifetime of the alcoholics studied. We previously reported increased microglial markers and the chemokine MCP1 in post-mortem human alcoholic brain [10]. These findings are consistent with gene array studies in post-mortem human brain. One of the most prominent gene groups altered in frontal cortex and VTA of alcoholics are 'immune/stress response genes' [53,54]. Similarly brain gene array studies in mice implicate pro-inflammatory genes in brain may as regulators of alcohol intake [55]. Thus, our findings are consistent with others.

Activated NOX produces superoxide. Superoxide formation, assessed by ethidine, was increased by ethanol. Increased NOX gp91 expression, superoxide formation in neurons (Figure 9) and increased makers of neuronal death (Figure 1, 2) are consistent with neuroimmune activation and oxidative stress mediating the neuronal toxicity.

Diphenyleneiodonium (DPI) inhibits NADPH-dependent oxidase. Our data found that co-treatment of DPI and ethanol significantly reduced ethanol induced microglial activation and ROS production (Figure 10, 11). Also, DPI pretreatment reduced ethanol increased caspase-3 immunoreactivity and Fluoro-Jade B staining (Figure 12). These data link NOX-ROS to ethanol-induced microglial activation and neurodegeneration.

**Figure 13:**
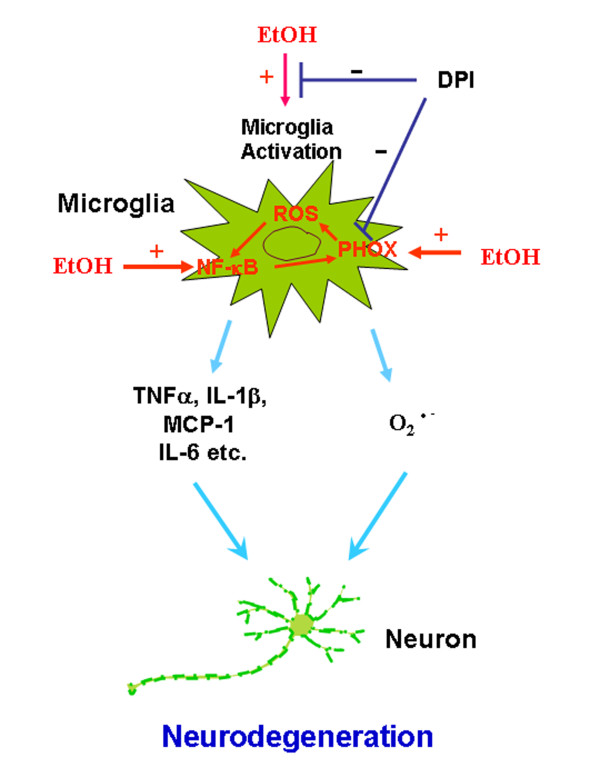
**NOX-ROS is a key signaling in alcohol neurodegeneration**. Alcohol as a pro-inflammatory trigger activates microglia to release neurotoxic factors, such as TNFα, IL-1β, MCP-1, IL-6, ROS (O_2_^-^). Among these pro-inflammatory factors, ROS have been implicated as key mechanisms of chronic neurotoxicity. Further, ROS upregulates NF-κB signaling, which causes microglial overactivation and propagate the cycle, driving progressive neuron damage. Blockade of NOX with DPI inhibits ROS generation, microglial activation and cell death markers: activated caspase-3 and Fluoro-Jade B.

This study supports a role of NOX and ROS in chronic ethanol-induced neuroinflammation and neurodegeneration (Figure 13). The present study and our previous report [9] find that chronic ethanol induces microglial activation, increases proinflammatory cytokines (TNFα, IL-1β, IL-6 etc.) and chemokines (MCP-1) and up-regulates NOX, resulting in production of ROS. NF-κB transcription is activated and generates these proinflammatory factors (cytokines, chemokines, oxidases, ROS) that amplify NOX-ROS and NF-κB signaling cascades (Figure 13). DPI, a NOX inhibitor, reduces microglial activation, ROS generation and neuronal death markers (activated caspase-3 and Fluoro-Jade B). Therefore, inhibition of NOX and ROS production may provide improved prevention and treatment for alcoholics and other neurodegenerative disorders.

## Conclusions

Chronic ethanol induces brain NADPH oxidase gp91^phox ^(NOX2) up-regulation and neurodegeneration in adult C57BL/6 mice that mimics findings in human alcoholic brain. Activation of microglia and astrocytes, induction of NOX and production of ROS contribute to ethanol neurodegeneration. Inhibition of NOX, ROS and NF-κB may offer hope in prevention and treatment for alcoholics and other neurodegenerative diseases.

## Abbreviations

ROS: Reactive oxygen species; PCR: Polymerase chain reaction; TNFα: Tumor necrosis factor-α; IL-1β: Interleukin-1β; MCP-1: Monocyte chemotactic protein-1; NF-κB: Nuclear factor kappa-light-chain-enhancer of activated B cells; IR: Immunoreactivity; ELISA: Enzyme-Linked ImmunoSorbent Assay; IACUC: Institutional Animal Care and Use Committee.

## Competing interests

The authors declare that they have no competing interests.

## Authors' contributions

LQ Participated in the experimental design, performed animal experiments and data analysis, and drafted the manuscript. FTC conceived the study, assisted with its design and data analysis, and helped draft the manuscript. All authors read and approved the final manuscript.
